# Use of Design of Experiments (DoE) to Model the Sulphate Agent Amount of (Ultra)Finely Ground and Fast Hardening Portland Cement Clinker

**DOI:** 10.3390/ma14195573

**Published:** 2021-09-25

**Authors:** Tim Schade, Bernhard Middendorf

**Affiliations:** Department of Structural Materials and Construction Chemistry, University of Kassel, 34125 Kassel, Germany; middendorf@uni-kassel.de

**Keywords:** design of experiments, Portland cement clinker, fine ground clinker, sulphate optimization, C-S-H-seeds

## Abstract

This paper presents a model to calculate the sulphate agent amount and sulphate agent ratio for fine grounded and fast hardening Portland cement clinker. Despite sufficient knowledge about the influence of calcium sulphate on the hydration process of cement, the sulphate agent amount is mostly adjusted empirically. As a result, often a wide and unfeasible experimental matrix has to be tested. In this work, Design of Experiments (DoE) was used in combination with in-situ X-ray diffraction (XRD) tests to accurately adjust the sulphate agent of different finely ground cement by calculation. With only 42 tests, it was possible to analyse in total the influence of the sulphate agent, the grinding fineness and the use of C-S-H-seeds for the use in fast-hardening Portland cement-based systems. In addition, it was found that a hemihydrate to anhydrite content of 25/75 leads to a stabilisation of the hydrated system in the first 24 h of hydration. A model for the optimisation of the sulphate agent composition in dependency of the cement fineness could be determined. Furthermore, it was shown that the DoE also provides optimal results in material sciences in a resource-saving way.

## 1. Introduction

### 1.1. Fast-Hardening Cements

The hydration of Portland cement is determined by the silicate and aluminate reaction. In the early hydration process, two decisive reactions take place partly in parallel and one after the other [1]:

(1) Silicate reaction of the clinker phases C_3_S (alite) and C_2_S (belite): Calcium silicate hydrates (C-S-H phases) and additional portlandite (Ca(OH)_2_) are formed. The hydration of the alite proceeds comparatively quickly. Although the tabular portlandite can be identified by X-ray, the C-S-H crystals on the contrary are apparently X-ray amorphous and thus can only be quantified to a limited extent by XRD analysis. High conversion of the silicate reaction stands for a high degree of hydration and high early strength.
(2) Aluminate reaction at the early hydration age of the C_3_A (tricalcium aluminate) with the sulphate agent: Prismatic ettringite and/or tabular monosulphate crystals are formed. In the absence of a sulphate agent, C-A-H phases are formed simultaneously. However, due to the mostly low crystallinity, the variable chemism and instabilities, a qualitative determination of the tabular monosulphate and the C-A-H phases is difficult [2].

According to Stark and Wicht [3], fast-hardening cements have a fast strength development. They are characterised by a short setting time and a high initial strength. Accordingly, all type of cement that set within one hour after mixing with water can be declared as fast-setting cements. The following possibilities are available to accelerate the reaction process:

- Increase in the C_3_S-amount
- Increase in the C_3_A-amount
- Change of sulphate agent and sulphate amount
- Increasing the grinding fineness of the cement
- Use of inorganic and organic additives.

Another way to accelerate the reactivity of the cements is to use alternative binders with geopolymer-like reaction properties. By adding aluminosilicate structures, a binary reaction consisting of cement hydration and geopolymer reaction takes place. This results in C-(N,K)-A-S-H phases incorporated with aluminium and alkalis [4]. In addition, other hydration products such as (N,K)-A-S-H phases, zeolites, hydroxysodalites or hydrotalcites can be formed, which contribute to the strength formation. Usually calcium-rich aluminosilicate or calcium hydroxide would induce rapid setting and high early strength [5,6]. Zhang et al. showed, for example, that by adding Bayer red mud, strength properties that normal cements reach after 28 days are already achieved after 7 days, see [7]. Finally, Portland cement-free blast furnace slag-based alkali-activated binders can also set rapidly and achieve high early strengths [8,9]. The choice of the alkaline activator plays a decisive role [10]. However, this paper focuses specifically on cementitious systems whose setting mechanisms are significantly influenced by the aforementioned clinker phases.

Typical applications of fast-hardening cements include various repair works (e.g., repair of pavements), shotcrete applications, tile adhesives and emergency measures (e.g., plugging of leaks to prevent water or other liquids from escaping). Alternative binder systems such as calcium aluminate cements, also called alumina cements (CA cements) and calcium sulfoaluminate cements (C$A cements) are often used for the above-mentioned applications. These fast-hardening inorganic binder systems that are currently investigated in the literature and used in current applications have a high aluminium and calcium sulphate content. The acicular hydration product ettringite is responsible for the rapid hardening behaviour at an early hydration age. Despite the fast-hardening process of CA cements, they show a critical drop in the final strength. This negative effect, known as conversion, has to be taken into account [11,12]. Moreover, C$A cements represent an environmentally friendly variant for rapid cements [13,14]. The main clinker phase of C$A cements is tetracalcium aluminate sulphate (C_4_A_3_$), also called Ye’elimite. Cements containing the Ye’elimite phase in combination with calcium sulphate also have a fast-setting behaviour and are suitable as fast cements due to their rapid strength development [15]. However, the strength development at the later hydration age depends on the calcium silicate content. Hence, C$A cements often have a poor durability and an undesirable expansion when used in the wrong dosage [16]. For this reason, various approaches exist for the production of C$A-belite cements [17,18]. 

Portland cement clinker with no/reduced sulphate agent are a fast-hardening binder alternative that has only been used occasionally so far. Usually, the strengths of mortars/concretes based on clinker powder without the use of a sulphate agent are lower compared to conventional Portland cements. Furthermore, these binders are not suitable for construction site.

Further possibilities to accelerate the hardening process are the use of finely ground clinker meal [19,20] and the use of C-S-H seeds [21,22,23], which have already been well tested. Due to a lack of applications and high costs, tests on very finely ground cements have only been analysed in a few investigations [24].

### 1.2. Models to Determine the Optimised SO_3_ Content in Dependency of Different Clinker Types

Finely ground Portland cement clinker with adapted sulphate agent represents an early strength-optimising alternative to organic or inorganic binder systems in the form of calcium sulphoaluminate or calcium aluminate cements. 

For the primary but also delayed primary formation of ettringite, the amount of C_3_A and the available sulphate agent supply is significant. An optimum sulphate content concerning the curing time is defined for clinker flours in various publications. The equations should help the user to set a standard-compliant setting and an optimal compressive strength after 28 days depending on the clinker powder used. In addition to a model by Haskell, Equation (1), there is another model for determining the optimal sulphate agent amount according to Jawed and Skalny, Equation (2) [3]:

SO_3,opt,Haskell_ = 0.0933 C_3_A + 1.7105 Na_2_O + 0.9406 K_2_O + 1.228 (1)

SO_3,opt,Skalny_ = 0.789 + 0.1149 C_3_A + 1.872 (Na_2_O + 0.658 K_2_O) (2)

However, the two models mentioned above do not include the grinding fineness of the clinker. A model according to Ost and Lerch [3] exists for calculating the optimal sulphate content for finely ground clinker. In this model, the grinding fineness of the clinker is taken into account by the Blaine value in cm²/g in the factor A_0_, see Equation (3):

SO_3,opt,Ost&Lerch_ = 0.5560 (Na_2_O + 0.658 K_2_O) + 0.0017659 A_0_ − 0.1072 Fe_2_O_3_ − 3.600 (3)

Since only the model according to Ost and Lerch involves the grinding fineness, the further experiments were determined as a function of the amount of SO_3_ considered optimal in Equation (3).

### 1.3. Design of Experiments (DoE)

In research and development, the structural organisation of experiments is at the beginning of the research work. Therefore, the organisation of experiments is the central point of successful experimentation. Within the scope of the investigations, essential differences and optima should be worked out by varying individual parameters [25,26,27]. Empirical experiments are always less suitable for predicting the properties, as in these experiments usually only a few of many possible parameters can be varied. The concept of Design of Experiments (DoE) is to increase efficiency by changing several components simultaneously. The aim should be to filter out as much information as possible with as few trials as possible. Due to the economic advantages of DoE, it is used in many branches of engineering [28,29], pharmaceutical [30] and chemical [31]. Most recently, the DoE has also shown promise for optimising the properties of inorganic materials. Nevertheless, it is not yet standard in building materials science [32,33]. 

The DoE can be set up in different ways; usually, the so-called D-optimal experimental design is used [34]. To achieve D-optimality, the determinant of the term (X’X)^−1^ in Equation (4) is minimised and consequently, the vector c is maximised:

c = (X′X)^−1^X′n_d_ (4)

In the DoE, the matrix X′X is called information matrix, so that by minimising the negative power, the information is maximised. In addition, there are further optimal experimental designs listed below [35,36]:

❖ A-optimality: optimisation of the mean-variance of the regression coefficients
❖ G-optimality: optimisation of the max. occurring variance of the prediction values
❖ V-optimality: optimisation of the mean prediction quality in the factor space.

For all experimental test plans, it is important to carry out a sufficient number of experiments to obtain a representative evaluation [26,27,37]. 

## 2. Materials and Methods

### 2.1. Basic Mixture

The used mixture in this study is based on the structural density-optimised Ultra High Performance Concrete (UHPC) formulation (M3Q) tested extensively in the priority programme (SPP) 1182 funded by German Research Foundation (DFG) [38]. In this study, the binder was mixed with a low w/b value of 0.3–0.45 depending on the fineness (Blaine-value) of the cement clinker:

- Clinker 3000 cm²/g (C3): w/b-ratio = 0.3
- Clinker 7000 cm²/g (C7): w/b-ratio = 0.35
- Clinker 12,000 cm²/g (C12): w/b-ratio = 0.45.

The three investigated clinker showed a comparable chemical composition. The results of the X-ray fluorescence (XRF) spectroscopy for each of the clinker meal is given in Table 1. Bulk chemical analysis was done by XRF at the Clausthal University of Technology, Institute of Non-Metallic Materials, Germany, in accordance to DIN EN 196-2.

As mentioned, the components influencing the early strength are the grinding fineness of the clinker meal, the sulphate agent composition and the use of hydration-accelerating additives and agents. In this study, the sulphate agent amount was divided into hemihydrate and anhydrite according to the experimental plan. In addition, the influence of C-S-H-seeds as an additive was analysed. The following binder components were used:

- Hemihydrate (7080 cm²/g Blaine)
- Anhydrite (7835 cm²/g Blaine)
- C-S-H-seeds (Circosil^®^ 0,1 (tobermorite), Circolit^®^ (xonotlite))
- Silica fume.

The active ingredient content of the superplasticizer (ViscoCrete-2810 with a solid content of 40 wt%) was 1.1. The mixture, especially the binder components were varied in the study depending on the DoE plan. Only the silica fume amount was fixed to 17.5 wt% and not taken into account. The individual components were calculated as given in Equations (5)–(7). The initials x_bws_ are an abbreviation for “binder without silica fume”. The total amount of SO_3_ in the binder system is composed of the three SO_3_-amounts: SO_3_ from XRF (see Table 1), SO_3_ hemihydrate, SO_3_ anhydrite:

(5) $$\mathrm{sulphate}\;\mathrm{agent}\;[\mathrm{kg}/m^{3}]=\frac{{\mathrm{SO}}_{3,\mathrm{opt},\mathrm{Ost}\&\mathrm{Lerch}}\;\cdot \;\left(x_{\mathrm{bws}}-\;C-S-H-\mathrm{seeds}\right)}{\frac{\mathrm{hemihydrate}\;\mathrm{amount}\;\left[\%\right]}{100\;\left[\%\right]}\;\cdot {\;\mathrm{SO}}_{3,\mathrm{hemihydrate}}+\frac{\mathrm{anhydrite}\;\mathrm{amount}\;\left[\%\right]}{100\;\left[\%\right]}\;\cdot \;SO_{3,\mathrm{anhydrite}}}$$

C-S-H-seeds [kg/m³] = C-S-H-seeds [%] ∙ x_bws_ (6)

Clinker meal [kg/m³] = x_bws_ − sulphate agent − C-S-H-seeds (7)

Subsequently, the samples are designated according to the following scheme:

➢ (clinker-fineness/1000)_(SO_3_[%])_(hemihydrate/anhydrite)

### 2.2. Analytics

#### 2.2.1. XRD

The X-ray phase analysis was carried out with an AXS D4 diffractometer from Bruker (Billerica, MA, USA) equipped with a LynxEye detector and the properties shown in Table 2. The analysis of the binder systems was carried out in situ. The phase change of the hydrating binder was detected every 10 min.

For the in-situ investigations, a plastic sample holder was used, which has a ring-shaped notch in the outer area. Thus, a foil can be stretched over the sample chamber by a matching ring. An X-ray-amorphous Kapton foil from Chemplex (Palm City, FL, USA) was used to largely avoid evaporation of the liquid during the measurement. The height errors that occur during in-situ sample preparation due to the Kapton foil, see also Hesse [39], were taken into account and corrected during the analysis.

#### 2.2.2. ESEM

A Quanta FEG 250 type environmental scanning electron microscope (ESEM; FEI, Hillsboro, OR, USA) was used for the scanning electron examinations. The scanning electron examinations of the raw material and the ground sections were carried out in low-vacuum mode using water vapour as ambient gas. The detailed investigations on the hydrating systems were carried out in high vacuum mode. For this purpose, small amounts of the binder were mixed and examined after one day. For analysis, fresh fracture surfaces of the samples were prepared and sputtered with gold/palladium in the Mini Sputter SC7620.

#### 2.2.3. Ultrasonic Test

The ultrasonic velocity was determined with a type IP-8 ultrasonic measuring system (UltraTest GmbH, Achim, Germany) and the UltraTestLab V2.0 user software. The fresh mortar was mixed with a hand mixer and filled into 130-20 LIV measuring moulds equipped with vibration dampers. The apparatus was specially made for measurements of the early setting process. The measurement was started during the filling process to track the setting time in-situ and to be able to conclude the setting behaviour.

### 2.3. Design of Experiments

Before the investigations, factors that influence the curing behaviour were defined for DoE, see Table 3. In addition, limits had to be selected for the factors according to a central composite design (CCD) [37]. As described in Section 1.2, the function according to Ost and Lerch takes into account the influence of the chemical composition and the grinding fineness of the clinker meals. However, the sulphate agent content, according to Equation (3), is only designed for good workability. It can be assumed that this sulphate agent content has to be reduced for optimising the strength and durability of fine clinker meals. Thus, with increasing fineness of grind, optimum workability and optimum strength are in conflict. Since for fast-setting clinker a prompt setting is desired, the optimisation of the strength was investigated in this study and a prefactor for the equation according to Ost and Lerch was determined. With this pre-factor, the equation for the stabilisation of the ettringite should be adjusted:

- The sulphate agent ratio was varied from 0/100–100/0 (hemihydrate/anhydrite).
- The sulphate agent amount was varied in defined steps by the choice of a prefactor concerning Equation (3). Without using a sulphate agent the mixtures did not allow processability.
- The amount of C-S-H-seeds was varied in relation to the cement content.
- The clinker meal was varied in the grinding finenesses 3000, 7000 and 12,000 cm²/g.
- The C-S-H-seeds type is divided into tobermorite- (Circosil^®^ 0.1) and xonotlite-structure (Circolit^®^).

## 3. Results

### 3.1. Development of a D-Optimal Test Matrix by DoE

A centrally composed response surface design was selected as the experimental design type to analyse interactions between the parameters. In addition, care was taken not to carry out any additional randomisation of the runs to ensure reproducibility of the DoE plan. Thus, only the randomisation automatically generated by Minitab^®^ (assignment of the experimental unit is not subject to any known random mechanism) was used.

In the further course, an attempt was made to keep the experimental runs to a minimum via D-optimality. The initial test plan was generated with the sequential method and improved with the Fedorov method. The named settings allow more possible experimental designs to be considered. The generation of an initial trial plan may take longer, but higher D-optima is set for the same number of points [37].

**Figure 1:**
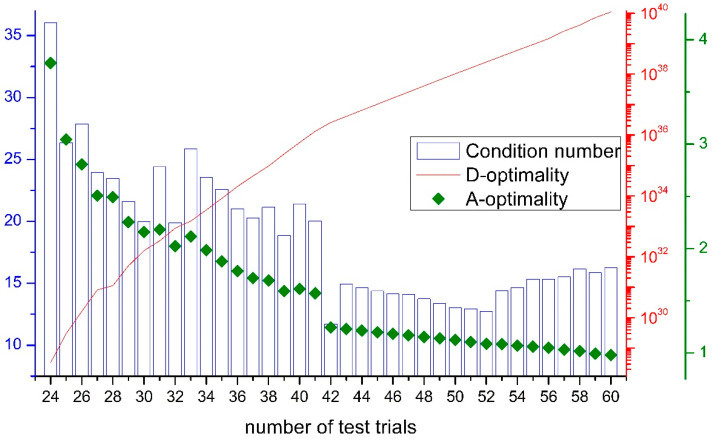
Analysis of the D- and A-optimality as well as the condition number in dependency of the test runs.

Figure 1 shows the calculated D-optimalities for different numbers of trial points, starting at 24 trial runs. It is noticeable that from 42 points onwards, a kink can be seen in the graph. In addition, the mean variance of the regression coefficients (A-optimality) also decreases sharply from a number of 42 trials. Consequently, the selected experimental design with 42 runs represents an experimental design selected according to D-optimality and at the same time A-optimality criteria. As mentioned, the respective optimality naturally increases with a higher number of experimental runs. As a third characteristic, the condition number can be used. The condition number provides information about the collinearity of the experimental design. Small collinearity is advantageous for the interpretation and accuracy of the results, as there are fewer correlations. In Figure 1, an optimum in the number of conditions can be found in 42 test runs, too.

### 3.2. Analysis of the Degree of Hydration with Regard to the Silicate Phase

**Figure 2:**
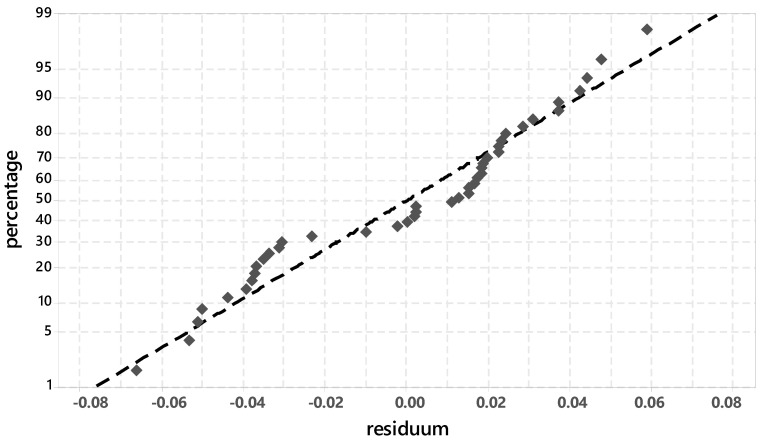
Probability plot for normal distribution for the degree of hydration phase (XRD-peak 32–32.5 °2ϑ).

Figure 2 shows the probability plot for normal distribution of the development of the degree of hydration from 0–24 h.

The main peak counts (mpc) of the C_3_S and the C_2_S in the range 32–32.5 °2ϑ were proportioned and values were determined for each sample. The background (bg) was subtracted in each case. The degree of hydration is determined according to Equation (8):

(8) $$\mathrm{Hydration}\;\mathrm{degree}\;[\%]=\frac{\mathrm{mpc}{\left(C_{3}S\;+{\;C}_{2}S\right)}_{24h}-\;\mathrm{bg}\;}{\mathrm{mpc}{\left(C_{3}S\;+{\;C}_{2}S\right)}_{0h}-\;\mathrm{bg}}$$

The individual test points are close to the adjusted line and thus indicate a normal distribution. With R² = 94.35%, a high probability can be assumed in the prognosis when determining the degree of hydration. 

**Figure 3:**
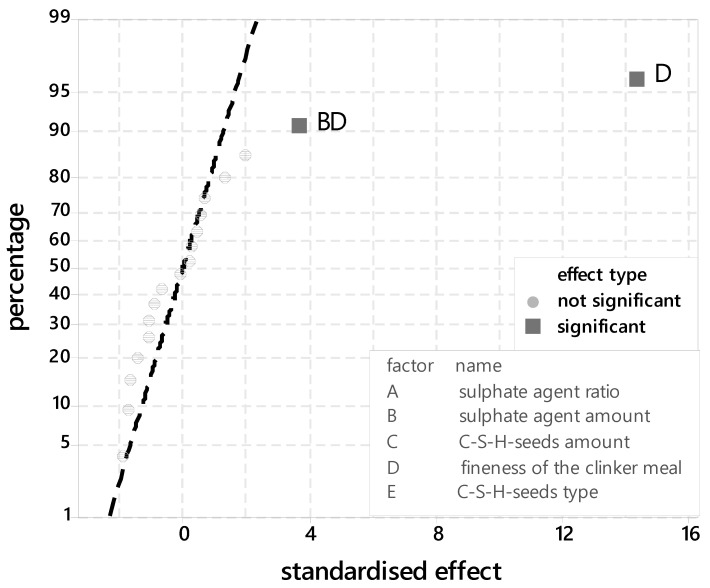
Probability plot for standardised effects for the silicate phase (XRD-peak 32–32.5 °2ϑ).

In addition, the probability network for standardised effects is shown in Figure 3. The significant parameters can be read. It is noticeable that the degree of hydration is significantly determined by the grinding fineness of the clinker.

**Figure 4:**
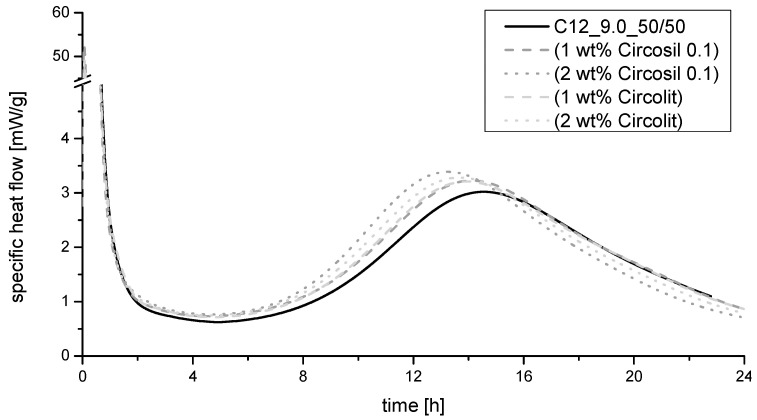
Specific heat flow measured for 24 h in dependency of C-S-H-seeds addition.

The addition of the two investigated C-S-H-seeds leads to a shortening of the dormant period and a slightly faster increase in heat flow during the acceleration period (measured by isothermal cement calorimetry, see Figure 4). The quantitative heat flow can also be classified as higher. Thus, it can be assumed that slightly more C_3_S is converted by the use of C-S-H-seeds in the investigated period. However, due to the defined investigation period and the strong dependence of the hydration on the grinding fineness, the use of C-S-H-seeds cannot be classified as a significant factor in this study.

### 3.3. Analysis of the Ettringite Formation with Regard to the Aluminum Phase

#### 3.3.1. Evaluation of Significant Factors Influencing the Ettringite Formation by DoE

Before the investigations, the curing time at which ettringite formation has a damaging effect was determined. It should be noticed that volume increases during plastic consistency do not lead to cracking as a result of deformation. For this purpose, ultrasound measurements were carried out and the time at a determined ultrasound speed of (1000 m/s) was defined as the critical curing point (CP). Due to the reduced sulphate agent content, the investigated samples showed immediate solidification for most of the mixtures within the first 10 min.

To detect the proportional change of the ettringite (C_3_A$_3_H_32_) formation in the system, the main peak counts of the ettringite in the range of approx. 9 °2 Theta were recorded. The main peak count was measured after 24 h of hydration and compared with the ettringite amount measured at the curing point (CP), see Equation (9). The production-related amorphous background (ug) from the Kapton foil was subtracted in each case:

(9) $$\mathrm{ettringite}\;\mathrm{change}\;[\%]=\frac{\mathrm{mpc}{\left(C_{3}{A\$}_{3}H_{32}\right)}_{24h}-\;\mathrm{ug}}{\mathrm{mpc}{\left(C_{3}{A\$}_{3}H_{32}\right)}_{\mathrm{CP}}-\;\mathrm{ug}}$$

**Figure 5:**
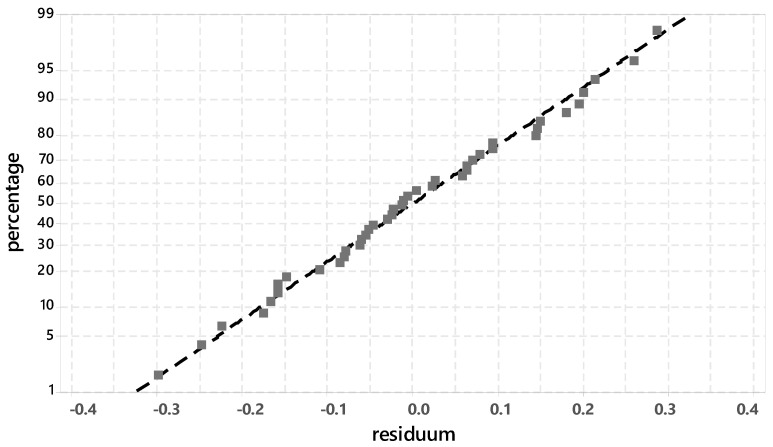
Probability plot for normal distribution for the aluminate phase (XRD-peak 9–9.5 °2ϑ).

The coefficient of determination was also in a very high range with R² = 97.13%. Therefore, it can be assumed that with the given variables the change of the ettringite peak over time (ettringite change) can be predicted precisely and the model is robust. The probability plot for normal distribution is shown in Figure 5.

**Figure 6:**
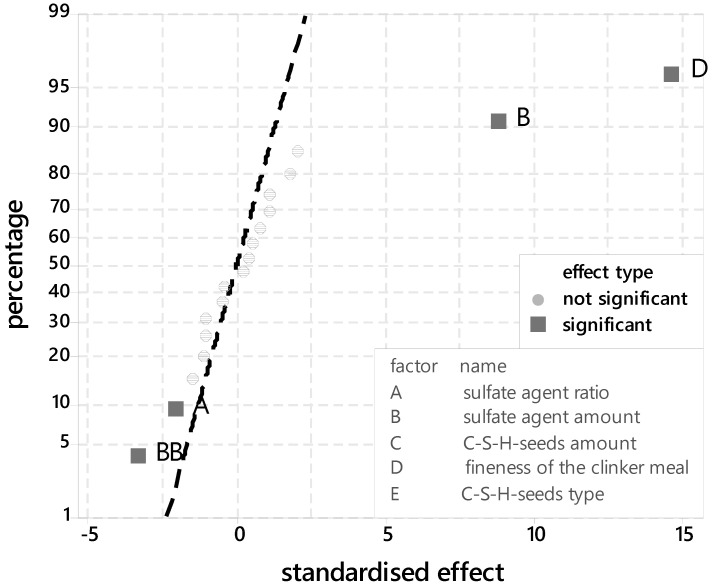
Probability plot for standardised effects for the aluminate phase (XRD-peak 9–9.5 °2ϑ).

The ettringite development in a time slot of 24 h depends on several factors shown in Figure 6. Comparable to the degree of hydration the grinding fineness is important for the aluminum reaction. Furthermore, the sulfate agent has a high impact on the ettringite development. In particular, the sulfate agent amount shows a quadratic effect.

#### 3.3.2. Chemical-Mineralogical Analysis of Phase Development 

**Figure 7:**
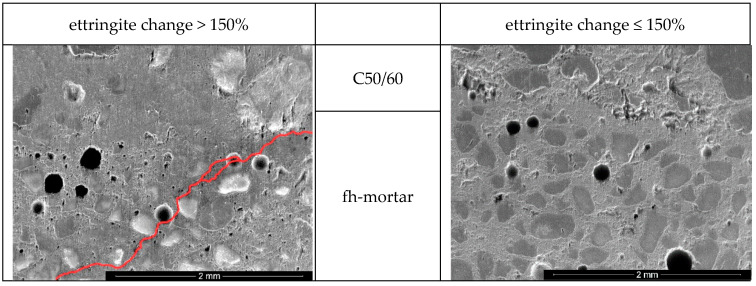
Characteristic ESEM pictures of samples with an 24 h-ettringite change of >150% and ≤150% (crack is marked).

To determine the value of ettringite change after 24 h that leads to cracking in the system, a standard concrete C50/60 was produced. A hole was drilled and the investigated mortars filled in. By this, possible ettringite expansion was observed. After 24 h of curing, sections were made and the bond area analysed by ESEM, see Figure 7. The investigated mortar is named fast hardening mortar (fh-mortar).

In the case of increased delayed primary ettringite formation (>150%), when the system had already solidified, cracking of the system occurred in each case. The cracks, which were only a few µm in size, extended from the investigated fresh mortar into the bonded area (see Figure 7; the crack was traced in red for better representation). 

It was also found that a slightly delayed primary ettringite formation of 140%, see Equation (4), led to a good bond and compacting effect.

**Figure 8:**
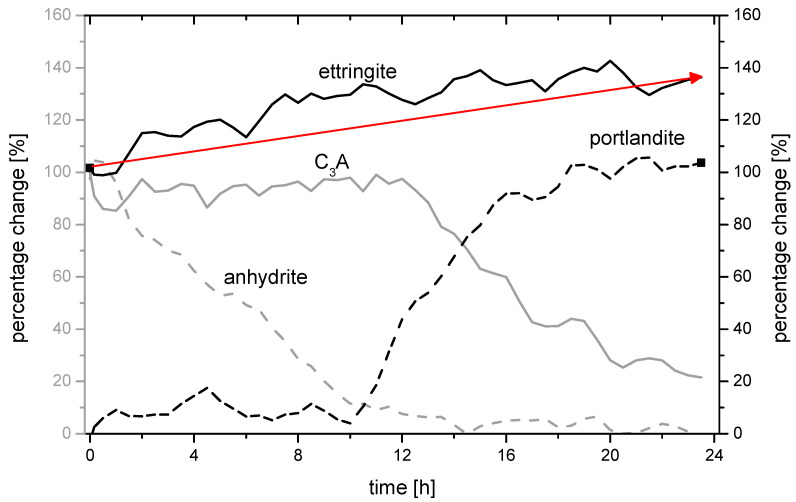
In-situ XRD-measurement of the mpc for different phases in sample C7_4.50_25/75.

**Figure 9:**
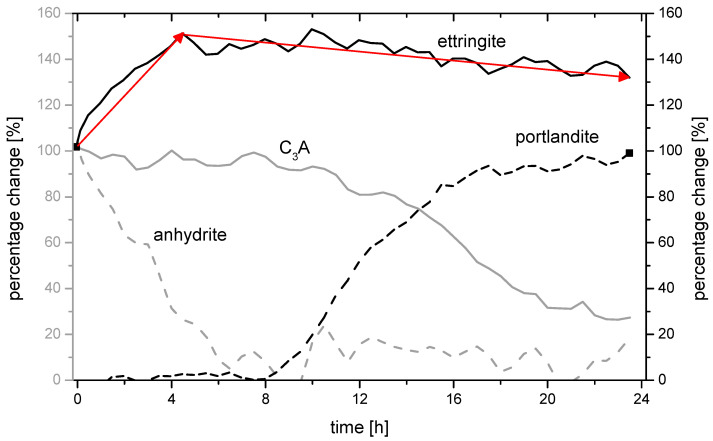
In-situ XRD-measurement of the mpc for different phases in sample C7_4.50_75/25.

Figure 8 and Figure 9 show the 24 h-changes in the ettringite formation measured by in-situ XRD in more detail. The following two samples are shown:

- C7_4.50_25/75 (ettringite change: 138.4%; 77.3 MPa)
- C7_4.50_75/25 (ettringite change: 132.9%; 72.5 MPa).

Both samples have comparable ettringite stability (130–140%). However, sample C7_4.50_25/75 with an increased proportion of anhydrite has a compressive strength of 77.3 N/mm², which is 4.8 N/mm² higher than sample C7_4.50_75/25 with an increased proportion of hemihydrate.

**Figure 10:**
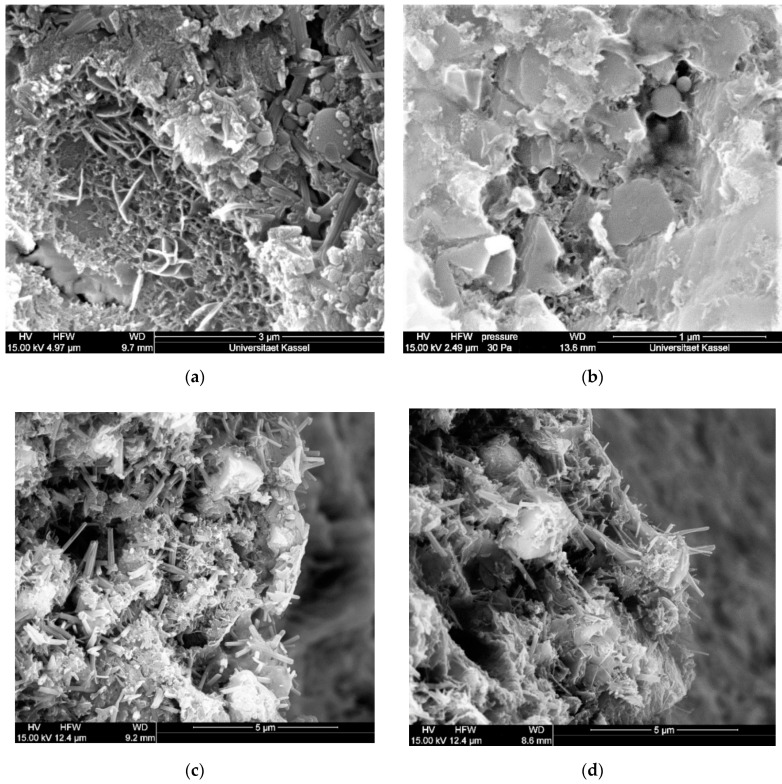
Monosulphate formation (**a**), C_3_AH_6_ (**b**) and ettringite formation (**c**) in sample C7_4.50_75/25 and ettringite formation (**d**) in sample C7_4.50_25/75.

The decrease of the C_3_A peak, as well as the increase of the portlandite peak, are comparable in both samples but start slightly earlier in sample C7_4.50_75/25. It can be assumed that both processes start after approx. 11 h. The development of the anhydrite content and thus the amount of ettringite in the system changes depending on the proportional composition of the selected sulphate agent. In the case of sample C7_4.50_75/25, the amount of ettringite increases until approx. 10 h after the mixing process and decreases in the course of hydration until approx. 20 h. At this point, the amount of C_3_A is also almost completely used up. The main ettringite peak in sample C7_4.50_25/75, on the other hand, increases almost constantly over time until about 20 h. The different dissolution rates of the sulphate agent components hemihydrate and anhydrite are responsible for the fact that different amounts of sulphate ions (SO_4_^2−^) are present in the system at different times and can react with the C_3_A to form ettringite and/or monosulphate, Figure 10a.

Accordingly, the stable phase C_3_AH_6_ was increasingly detected in sample C7_4.50_75/25 after 24 h, Figure 10b. It was also observed that the ettringite phases (Figure 10d) from the anhydrite were smaller compared to the ettringite phases that developed primarily and predominantly from the hemihydrate (Figure 10c).

**Figure 11:**
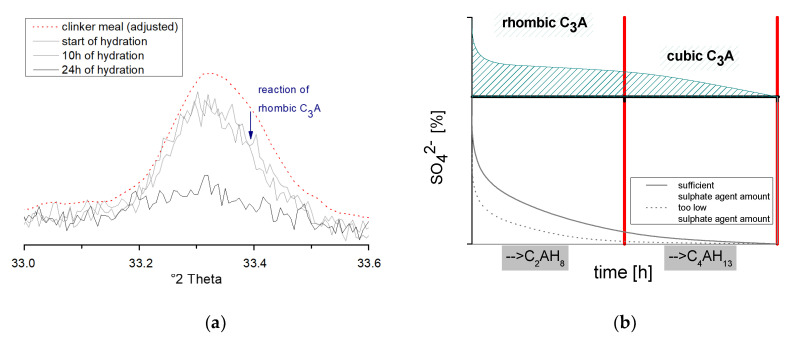
XRD development of C_3_A peak within 24h of hydration (**a**) and schematic development of cubic and rhombic C_3_A (**b**).

In addition, the described reaction process could be detected using in-situ XRD investigations. By matching the unhydrated clinker (measured by backloading) to the peaks of the freshly prepared and hydrating sample, it could be observed in each case that the C_3_A peak initially showed a decrease in the larger °2ϑ range in Figure 11b. For example, Dubina et al. [40] showed that the rhombic modification has the maximum peak in slightly higher angular regions in the range 33.4 °2ϑ. After about 10 h of hydration, the second dissolution phase of C_3_A starts (33.2–33.3 °2ϑ). At this point, predominantly the cubic C_3_A and to a minor extent, the remaining rhombic C_3_A is further converted (see Figure 11a).

## 4. Discussion and Evaluation

### 4.1. Modeling the Optimal SO_3_-Content for Clinker Meals with a High Grinding Fineness 

From the results of the 42 samples investigated (14 per grinding fineness), equations for determining the optimum ettringite change as a function of the components sulphate agent content (Xs_content_) and sulphate agent ratio (Xs_ratio_) can be determined for all 3 grinding finenesses (3000 cm²/g = C3, 7000 cm²/g = C7, 12,000 cm²/g = C12). As explained beforehand, the optimal range was defined at an ettringite change of 140%. Subsequently, it is possible to model the following Equations (10)–(12) depending on the grinding fineness:

C3: ettringite-change = 1.066 × X_Scontent_ − 0.00255 × X_Sratio_ − 0.404 (10)

C7: ettringite-change = −1.497 × X_Scontent_² + 3.311 × X_Scontent_ − 0.00255 × X_Sratio_ + 0.183 (11)

C12: ettringite-change = −1.497 × X_Scontent_² + 3.311 × X_Scontent_ − 0.00255 × X_Sratio_ + 0.413 (12)

It is noticeable that only the sulphate agent fraction (X_Scontent_) shows a quadratic effect in the system. For the coarse grinding fineness C3, a linear equation results. This linearity can be justified by the fact that the equation according to Ost and Lerch is not suitable for coarse finenesses of clinker meal; thus, the sulphate agent content was too low in each mixture.

The microscopic investigations have shown that a moderate ettringite increase does not allow microstructural cracks and, on the contrary, densifies the microstructure. In addition, this small increase in ettringite formation excludes a regressive transformation to CAH phases and early CAH phase formation. Based on the results obtained in Section 3.3.2, it can be assumed that in addition to a sulphate agent ratio of 25% hemihydrate to 75% anhydrite, the change in ettringite after 24 h should be in the range of 135–145%. By rearranging the Equations (10)–(12) and inserting 25 for X_Sratio_ and 1.4 (140%) for the ettringite change, the following optima for the prefactor for calculating the optimal sulphate agent amount in the system are obtained:

C3: X_Scontent_ = 1.75
C7: X_Scontent_ = 0.50
C12: X_Scontent_ = 0.39.

The main goal in modelling the sulphate agent amount of clinker meals with high grinding fineness was to create a model for the user that consists of a single overall equation. The overall equation can be well represented by a 2D-parabolic curve in which the sulphate agent fraction shows a quadratic influence. To model the determined measured values as accurately as possible by an overall function, a function is needed that has the following properties:

The function:

❖ runs through the measured values determined using DoE
❖ is monotonically decreasing in the interval I_1_ = [3; ∞)
❖ only slowly tends towards 0 for x → +∞
❖ has exclusively positive y-values in the interval I_1_ = [3; ∞).

According to the properties mentioned, the following function types are possible:

❖ exponential function of the form f_1_(x) = a∙e^bx^, f_2_(x) = b∙a^kx^
❖ power function of the form f_3_(x) = a∙x^n^ (n ∈ R), which guarantees monotonicity as written above
❖ fractional rational function of the form f_4_(x) = $\frac{\mathrm{ax}}{x-b}$ und f_5_(x) =$\;\frac{\mathrm{ax}}{x-b}+c$.

If the values do not approximately correspond for any of the functions mentioned, a piecewise defined function is also conceivable. The following values are defined as measured values (grinding fineness in thousands):

f_k_(3) = 1.75
fk(7) > k(7) = 0.50
fk(12) k(12) = 0.40 (rounded up).

If an attempt is made to model the points by one of the named function types with the help of the open-source programme GeoGebra, it is immediately apparent that the function types of the exponential (f_1_(x), f_2_(x)) and power function (f_3_(x)) cannot represent the required values accurately enough.

With the help of the substitution method, the solution for the fractional rational function can be determined mathematically. Since the function f_5_(x) contains three parameters, the system of equations is, therefore, neither underdetermined nor overdetermined; this is to be preferred. To simplify matters, the solution was determined using the computer algebra system “TI-NspireTM CX CAS Teacher software”. An equation with the following parameters is modelled as the solution for the fractional rational function:

a = 0.372208
b = 2.38462
c = 0.064516.

Equation (13) results as a functional equation. The variable x (Blaine-value/1000) can also be defined as A_0_ and accordingly stands for the Blaine-value of the clinker flour in cm²/g:

(13) $$f_{5}(x)\;=\;f_{5}(A_{0})\;=\;\frac{0.372208\;\cdot {\;A}_{0}\;/\;1.000}{A_{0}\;/\;1.000-2.38462}-0.064516$$

As mentioned, this function is to be understood as a prefactor of the function according to Ost and Lerch. With the help of this equation, the function for determining the optimum SO_3_ content according to Ost and Lerch concerning the setting time should be adapted for fast-setting Portland cement-based systems. At the same time, no damaging delayed primary ettringite occurs in the system at the resulting SO_3_ content.

The optimum SO_3_ content (f_compound_(Na_2_O, K_2_O, A_0_, Fe_2_O_3_)) for the production of inorganic composite mortars with high clinker fineness is shown in Equation (14). The equation is a compilation of the prefactor from the DoE (f_5_(A_0_)) Equation (13) with the equation according to Ost and Lerch Equation (3):

(14) $$f_{\mathrm{compound}}({\mathrm{Na}}_{2}O,\;K_{2}O,\;A_{0},\;{\mathrm{Fe}}_{2}O_{3})\;=\;f_{5}(A_{0})\;\times \;{\mathrm{SO}}_{3,\mathrm{opt},\mathrm{Ost}\&\mathrm{Lerch}}\;=\;\frac{0.372208\;\cdot \;\frac{A_{0}}{1000}}{\frac{A_{0}}{1000}-2.38462}-0.064516\cdot \left(0.5560\left({\mathrm{Na}}_{2}O+0.658\cdot K_{2}O\right)+0.0017659\cdot A_{0}-0.1072\cdot {\mathrm{Fe}}_{2}O_{3}-3.600\right)$$

**Figure 12:**
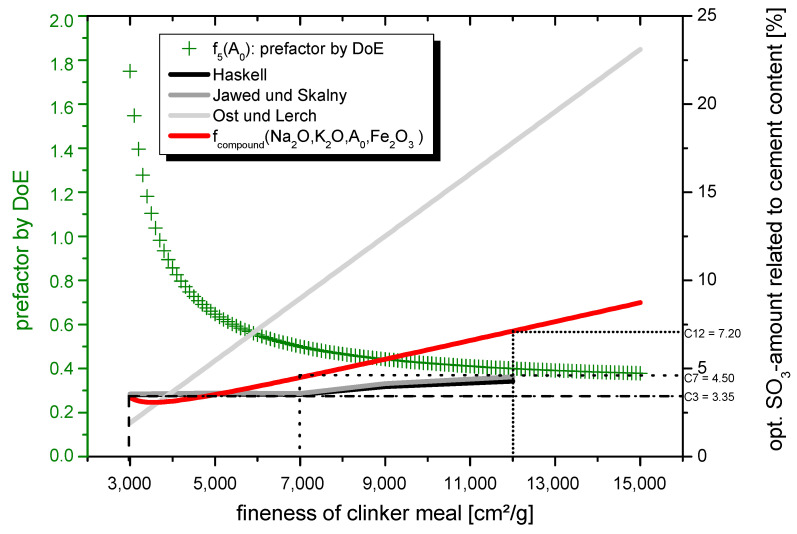
Diagram to determine the optimum SO_3_-amount in dependency of the grinding fineness of clinker meal.

The equations are shown graphically in Figure 12. 

With the help of a suitable hemihydrate/anhydrite ratio, CAH phase formation is also avoided. However, it must be taken into account that the sulphate agent ratio may vary slightly when choosing a different cement clinker depending on the rhombic and cubic fraction of the tricalcium aluminate. In this case, the equation has to be adjusted.

### 4.2. Evaluation

According to the equation for the material model, optimal mixtures can be set for the coarse and fine clinker grinding fineness depending on the sulphate agent supply. Within the framework of evaluation tests, it is checked whether the optima calculated according to the model also show the best compressive strength values after 24 h.

It could be shown in Section 3.3.2 that a sulphate agent amount of 4.50% SO_3_ for the clinker meal with a grinding fineness of 7000 cm²/g is suitable. A non-damaging primary as well as delayed primary ettringite formation in the value range of (135–145%), i.e., a moderate increase of the ettringite content in the system due to delayed primary ettringite formation, is to be assumed. As already explained, this moderate increase in ettringite is equivalent to an avoidance of the strength-reducing C_3_AH_6_ phase. The following values result for the coarse and (ultra)fine clinker meal by insertion into Equation (14) (values of the chemical composition see XRF, in Section 2.1.):

f_compound_ (Na_2_O, K_2_O, 3000, Fe_2_O_3_) = 3.35% SO_3_
f_compound_ (Na_2_O, K_2_O, 12,000, Fe_2_O_3_) = 7.20% SO_3_.

**Figure 13:**
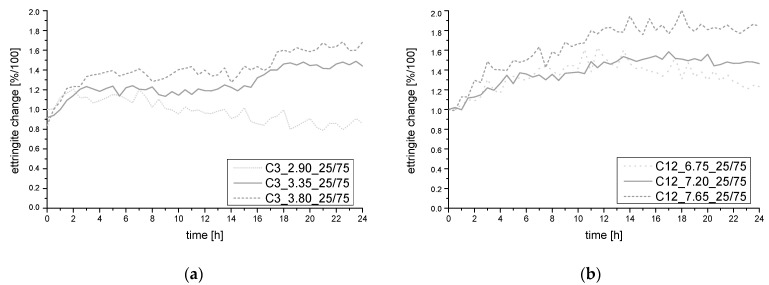
Change of ettringite main peak count for the clinker with 3000 cm²/g (**a**) and 12,000 cm²/g (**b**).

To verify the test results, in-situ XRD investigations were carried out again, see Figure 13. Therefore, the coarse and fine clinker meals were investigated. The optimum ettringite formation development with the calculated SO_3_ contents from the developed model could be confirmed. 

In addition, compressive strength values were determined on 2 cm cubes after 24 h hydration to check the strength development. All samples were tested three times and the mean value was calculated. The results are shown in Table 4. For both compressive strengths determined, the optima could be set in the determined range.

## 5. Conclusions and Outlook

In this work, a prediction model to determine the optimum SO_3_ content in dependency of the grinding fineness concerning an optimum in strength could be developed. The sulphate agent content along with a suitable sulphate agent ratio are considered to be the control variables for the adjustment of the fresh and hardened concrete properties in the investigated fast hardening systems. The work showed that the optimum sulphate agent content has to be adjusted when the grinding fineness is changed. An increase in the SO_3_ content is mandatory with increasing grinding fineness of the clinker meal. The considerations of Ost and Lerch [3] for the determination of the optimum SO_3_ content for higher grinding finenesses are to be classified as correct about the setting time. Only by adjusting the SO_3_ content in the model developed in this work, it is possible to stabilise the formation of ettringite for 24 h and thus avoid delayed primary ettringite formation and undesirable CAH phase formation in the binder system. The deviation of the model is based on the curing point (CP). The CP was determined by means of an ultrasonic test to calculate the ettringite change over time. Consequently, the procedure/technology described in this paper is only applicable for faster curing cements that set within one hour as stated in the definition of rapid hardening cements. In addition, it should be taken into account that the developed model is applicable to sulphate-supported fast cements. Consequently, fast hardening cements that are not based on early strength formation by ettringite are not included in this model.

It should also be noted that adjusting the optimum sulphate agent content as a function of the grinding fineness of the clinker meal leads to setting times that vary in speed. For fast-setting clinker systems, the use of a higher fineness of grind is therefore unavoidable. However, the compressive strength is not necessarily due to the faster reactivity, as assumed beforehand, since all optimised samples show high compressive strength values regardless of the grinding fineness in the system.

It could be shown that an SO_3_ ratio with a higher proportion of anhydrite and not with the more soluble hemihydrate leads to more favourable strength development. Nevertheless, it should be noted that an adjustment is necessary depending on the percentage ratio of rhombic to cubic C_3_A. On the contrary, it could be shown that the formation of fine ettringite needles from the cubic C_3_A reaction makes a significant contribution to strength. In the presence of Ca(OH)_2_, thin and elongated ettringite crystals were formed, which led to additional interlocking in the binder system.

## Figures and Tables

**Table 1:** XRF-results for the three used clinker meals C3, C7, C12.

Oxide	C3 (Clinker 3000 cm²/g)	C7 (Clinker 7000 cm²/g)	C12 (Clinker 12,000 cm²/g)
	[wt%]	[wt%]	[wt%]
SiO_2_	22.00	20.88	19.77
Al_2_O_3_	5.90	5.59	5.73
Fe_2_O_3_	2.14	2.13	2.37
CaO	66.94	65.74	63.85
MgO	0.97	0.92	0.97
SO_3_	0.82	0.80	1.43
Na_2_O	0.37	0.34	0.45
K_2_O	0.65	0.66	1.16
Cl	0.01	0.01	0.02
P_2_O_5_	0.14	0.14	0.13
sulphide	0.09	0.10	0.14

**Table 2:** XRD-properties used in this study.

Parameter	Value
Radiation	CuKα
Voltage/Current	40 kV/30 mA
Angle range	5° 2 Theta–65° 2Theta
Step width	0.0057 °2Theta
Measuring time per step	0.05 s
Total number of steps	10,522
Delay time	47 s
Measuring time per range	10 min
Divergence gap	0.05 mm

**Table 3:** Influencing factors to accelerate the hardening process that were varied by DoE in this study.

Factor	Type	Bottom Star Point	Lower Limit	Center Point	Higher Limit	Upper Star Point
Sulphate agent ratio	steady	0/100	25/75	50/50	75/25	100/0
SO_3_-amount (pre-factor Ost & Lerch)	steady	0.25 *	0.5 *	0.75 *	1.0 *	1.25 *
C-S-H-seeds amount	steady	0 wt%	0.5 wt%	1 wt%	1.5 wt%	2 wt%
Fineness of clinker	categorical		3000 cm²/g	7000 cm²/g	12,000 cm²/g	
C-S-H-seeds type	categorical		Circosil^®^ 0.1		Circolit^®^	

*: prefactor.

**Table 4:** Compressive strength values for the clinker with coarse and fine grinding fineness.

Clinker 3000 cm²/g		Clinker 12,000 cm²/g	
C3_2.90_25/75	67.3 MPa	C12_6.75_25/75	70.7 MPa
C3_3.35_25/75	71.4 MPa	C12_7.20_25/75	76.2 MPa
C3_3.80_25/75	63.1 MPa	C12_7.65_25/75	72.1 MPa

## Data Availability

The data presented in this research study are available in this article.
